# A chloroplast genome of *Forsythia saxatilis* (Nakai) Nakai, an endemic species in Korea

**DOI:** 10.1080/23802359.2020.1834889

**Published:** 2020-11-13

**Authors:** Sang Chul Choi, Sangtae Kim

**Affiliations:** Department of Biotechnology, Sungshin University, Seoul, Korea

**Keywords:** Chloroplast genome, endangered species, *Forsythia saxatilis*, Oleaceae

## Abstract

*Forsythia saxatilis* (Nakai) Nakai is an endemic species in Korea. We present the second complete chloroplast genome sequence of *F. saxatilis* showing that the chloroplast genome is 156,376 bp in length containing four subregions: a large single-copy (LSC) region of 87,097 bp, a small single-copy (SSC) region of 17,859 bp, and a pair of inverted repeat regions (IRs) of 25,710 bp. The genome contains 131 genes including 88 protein-coding genes, 35 *tRNA*s, and eight rRNAs. We found 40 base differences in 17 sites between our chloroplast genome and the previously reported chloroplast genome sequences of *F. saxatilis*. All of the differences were found in a region of 832 bp in the LSC region except for one site, which indicated potential sequencing errors in building the previously reported genome because of little substitutions in the homologous region of the other *Forsythia* species and even of *Abeliophyllum distichum*, a sister to *Forsythia*. The new chloroplast genomes of *F. saxatilis* and those of *F. x intermedia* and *F. viridissima* are identical, which suggests that *F. saxatilis* is maternally closely related to the other two species.

*Forsythia* Vahl (Oleaceae) contains 13 species distributed mostly in eastern Asia except for one species in southeastern Europe (Ha et al. 2018). Species of *F. koreana* (Rehder) Nakai, *F. nakaii* (Uyeki) T. B. Lee, *F. ovata* Nakai, and *F. saxatilis* (Nakai) Nakai have been reported as endemic species in Korea (National Institute of Biological Sciences 2013). In particular, most distribution of *F. saxatilis* is in the Bukhansan National Park in Korea as an endangered and flagship species of the park (Kim and Chang 2007). Additional fragmented distributions of *F. saxatilis* are found in limestone area of Gangwon province and Euysung-gun in Gyungbuk province (Han et al. 2011).

The first chloroplast (cp) genome from *F. saxatilis* has been reported in the phylogenetic study of tribe Forsythieae in Oleaceae (Ha et al. 2018). In this study, we found a new population of *F. saxatilis* in Opaesan (N20°27′55.14′′, W105°17′31.21′′) where is near from the Bukhansan National Park and determined the second cp genome with a sample from the new population. It provides comparative genomic evidence that the first cp genome could be affected by potential sequencing errors.

We collected a plant and leaves were preserved in silica gel. A voucher specimen was deposited in the herbarium of the Sungshin University, Korea (*S. Kim 2020-001*, SWU). DNA extraction was performed using a commercial plant DNA extraction kit (Exgene^TM^; GeneAll, Seoul, Korea), and the extracted DNA was sequenced using the MGISEQ-2000 platform (MGI Tech Co. Ltd., Shenzhen, China) with paired-end reads (150 bp in length). The sequencing library was constructed using the MGIEasy DNA library Prep Kit (MGI Tech Co. Ltd., Shenzhen, China) with an insert size of 500 bp.

The sequencing generated a total of 20,477,062 raw reads. After short-read quality filtering, the reads were mapped against the previously reported cp genome from *F. saxatilis* (MF407179) using Geneious version 9.1.8 (Kearse et al. 2012). We also performed *de novo* assembly of the mapped reads using Geneious, which produced the same sequence as the mapped result. Comparing our cp genome with the previously reported one from *F. saxatilis*, we found 40 base differences in 17 sites. The sites included two short repeating unit differences (TTATAAA and ATGCTA), two three-concatenated base changes, two homopolymer indels, three homopolymer size differences, and eight single-nucleotide substitutions between the two cp genomes. Remarkably, all of the differences except for one site were found in the intergenic region between *aptF* and *atpH* and parts of the two genes, which was a relatively short region (832 bp) of the large single-copy (LSC). Furthermore, there were not so many substitutions in the region of the other *Forsythia* species and even of *Abeliophyllum distichum,* a sister to *Forsythia* in Oleaceae (Ha et al. 2018). Therefore, we believe that this region in the previously reported cp genome from *F. saxatilis* is likely to be affected by potential sequencing errors.

The assembled cp genome (MT887608) was annotated using Geneious version 9.1.8 (Kearse et al. 2012) based upon the annotation of *F. mandschurica* (NC_048504.1), one of the reference cp genomes in *Forsythia*. The size of the cp genome of *F. saxatilis* was 156,376 bp, consisting of a LSC region of 87,097 bp and a small single-copy (SSC) region of 17,859 bp separated by each of the two identical inverted repeat regions (IRs) of 25,710 bp. The cp genome encoded 88 protein-coding genes, 35 tRNAs, and eight rRNAs.

A phylogenetic analysis was performed using (1) the new cp genome of *F. saxatilis*, (2) 15 cp genomes of *Forsythia* species, and (3) the cp genome of *Abeliophyllum distichum* Nakai (NC_031445) (Figure 1). These 17 sequences were aligned by the MAFFT program (Katoh and Standley 2013) and a maximum-likelihood analysis was performed to create a phylogenetic tree using IQ-TREE (Nguyen et al. 2015) with the best model (K3Pu + F + I) including 1000 replicates of bootstrapping.

**Figure 1.**
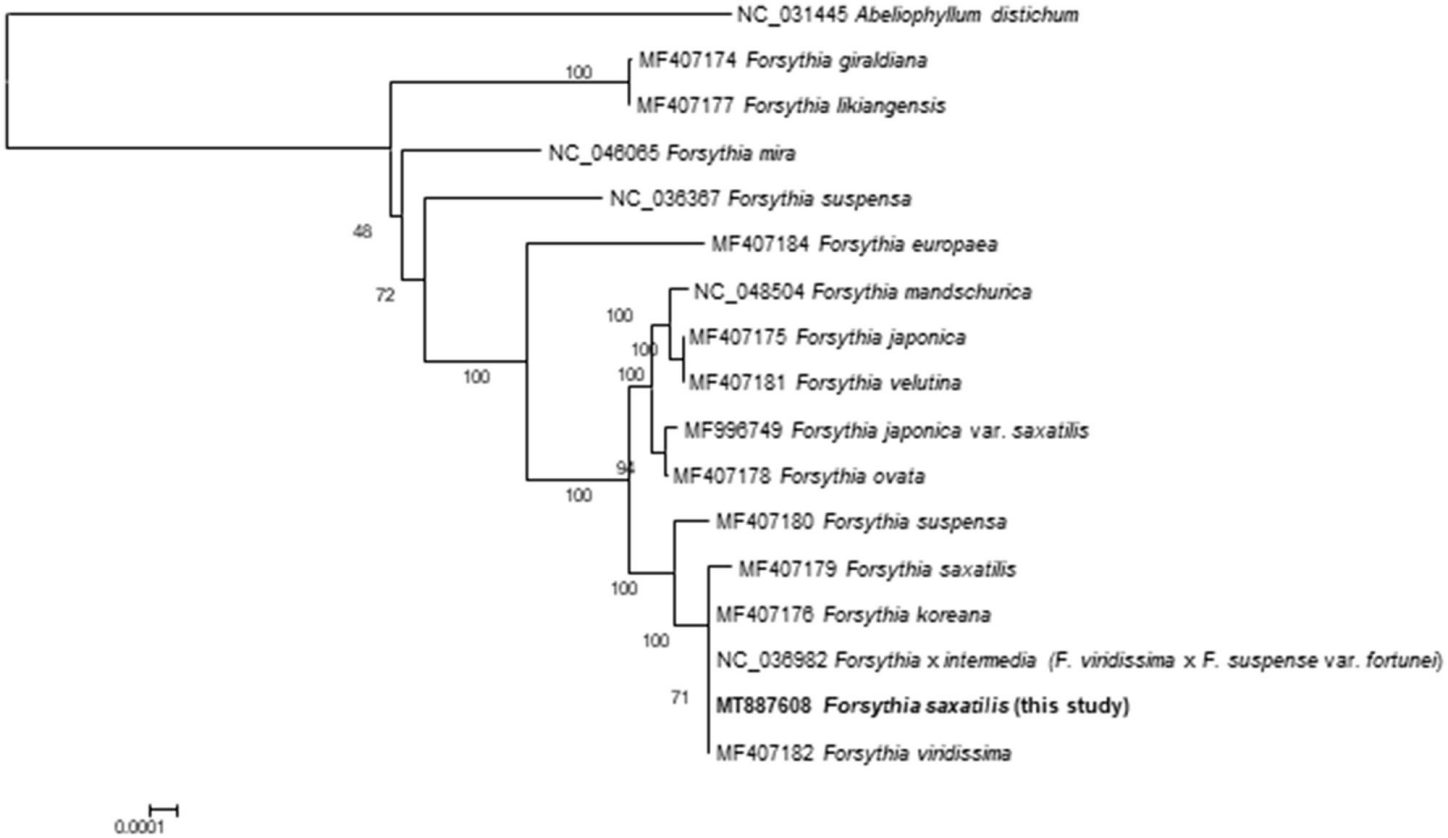
A maximum-likelihood tree based on an alignment of entire cp genome sequences that are from *F. saxatilis*, 15 species of *Forsythia*, and *Abeliophyllum distichum* as an outgroup.

The tree showed that our cp genome was clustered with those of *F. koreana* (MF407176), *F.* × *intermedia* (= *F. viridissima × F. suspense* var*. fortunei*) (NC_036982), and *F. viridissima* (MF407182) (Figure 1). The previous cp genome of *F. saxatilis* (MF407179) was more distantly related to them than our cp genome. The new cp genome sequence of *F. saxatilis* was exactly the same as those of *F.* × *intermedi* and *F. viridissima*, and differed only in two bases from that of *F. koreana.* Autapomorphic characters found in the previous cp genome of *F. saxatilis* probably due to potential sequencing errors resulted in the rather longer external branch leading to the previous cp genome of *F. saxatilis*.

We report the second complete chloroplast genome in *F. saxatilis.* This allowed comparison with the previously reported cp genome in the species, suggesting the possibility of sequencing errors. The base substitution rate and phylogenetic relationship among species in *Forsythia* provided by this study elucidate the evolutionary history such as a hybridization origin.

## Data Availability

The data that support the findings of this study are openly available in NCBI at https://www.ncbi.nlm.nih.gov/ (reference number: MT887608) or available from the corresponding author.
